# Superficial acral fibromyxoma with bone involvement: description and postoperative follow-up^☆^

**DOI:** 10.1016/j.abd.2024.04.015

**Published:** 2025-01-09

**Authors:** Ana Carolina Baião Silva, Helena Maciel Guerra, Leonardo Ávila Ferreira, Rafael Fantelli Stelini, Laura Bertanha, Renata Ferreira Magalhães

**Affiliations:** a Department of Clinical Medicine, Faculty of Medical Sciences, Universidade Estadual de Campinas, Campinas, SP, Brazil; b Department of Pathology, Faculty of Medical Sciences, Universidade Estadual de Campinas, Campinas, SP, Brazil; c Dermatology Service, Hospital do Servidor Público Municipal de São Paulo, São Paulo, SP, Brazil

*Dear Editor,*

Superficial acral fibromyxoma (SAF) is a rare mesenchymal tumor with slow growth, fibroelastic consistency, and is generally painless. It mainly affects the hands and feet, particularly the periungual or subungual region. It is more common in men, with a ratio of 2:1, occurring around the age of 50.^1^

This tumor exhibits polymorphic features, making its diagnosis challenging. When located in the periungual region, it typically presents as a whitish or pinkish mass with mild hyperkeratosis and no visible vascular structures. On the other hand, when situated in the subungual region, it causes lunula deformity. In these cases, microhemorrhages and dilated linear vessels can be visualized on dermatoscopy.^2^, ^3^

We report a case of SAF with bone involvement, detailing its dermatoscopic, histological, and evolutionary aspects.

A 71-year-old woman reports the appearance of a painless tumor on the left fourth toe with progressive growth for the past 5 years. On physical examination, she exhibited an erythematous lesion with fibroelastic consistency, crusts, telangiectasias, and a collar at the base (Fig. 1 A and B). Dermatoscopic examination revealed dilated linear vessels, brownish amorphous areas, and compact white plaques, suggestive of altered keratinization (Fig. 1 C and D).

**Fig. 1:**
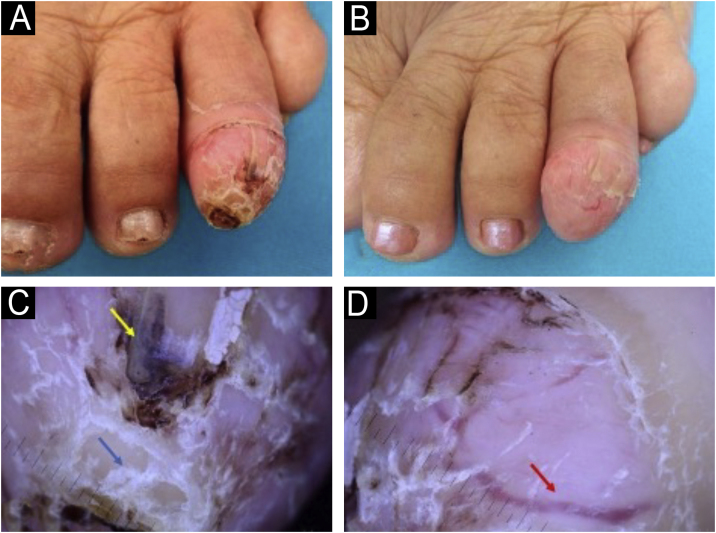
(A) Keratotic tumor on the left fourth toe. (B) 14 days after photo A ‒ Growing tumor with more evident thinning of the nail plate and telangiectasias on the surface. (C and D) Dermatoscopy 10× Presence of dilated linear vessels (red arrow), brownish amorphous areas (yellow arrow), and compact white plaques (blue arrow).

On the radiograph, bone erosion associated with the thickening of soft tissues in the distal phalanx of the left fourth toe was evident (Fig. 2). An excisional biopsy without margins was performed for histological diagnosis.

**Fig. 2:**
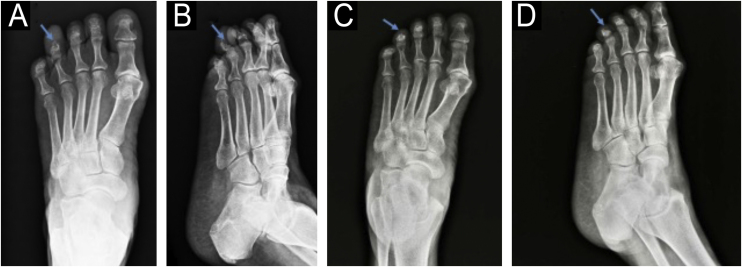
Anteroposterior (A) and lateral (B) radiographs of the left foot before the surgery: increased soft tissue density and bone erosion of the distal phalanx (blue arrow), resembling a “cup” appearance (B). Anteroposterior (C) and lateral (D) radiographs of the left foot two months after the surgery: partial recovery of bone structure.

The histopathological examination revealed dermal spindle cell proliferation amidst myxoid stroma (Fig. 3 A and B). Immunohistochemical examination showed positivity for CD34 (Fig. 3C), CD99 (Fig. 3D), and Ki67 in less than 5% of cells and negativity for 1A4, S100 and epithelial membrane antigen (EMA).

**Fig. 3:**
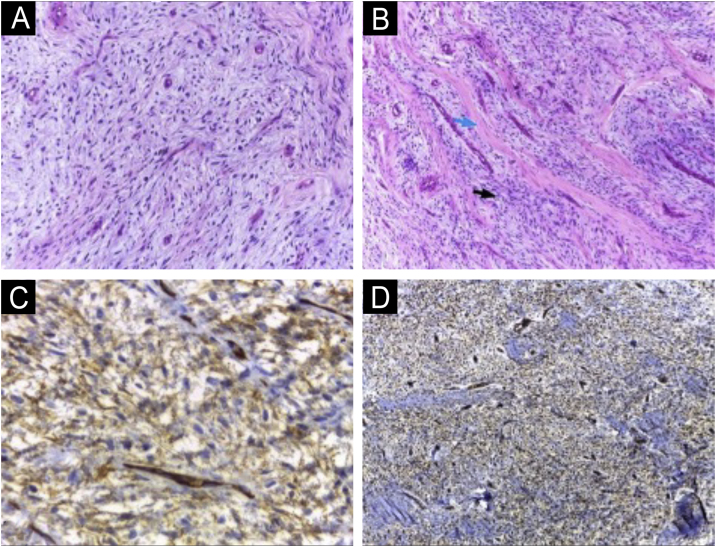
(A and B) Homogeneous spindle cell proliferation amidst myxoid stroma (A); fibrosis areas (blue arrow) alternating with myxoid stroma (black arrow) (B) (Hematoxylin & eosin; 100×). Immunohistochemical study showing neoplastic cells with diffuse expression of CD34 (C) and CD99 (D) (400× and 100×, respectively).

Given the clinical presentation, histopathological, and immunohistochemical aspects, SAF was diagnosed. The patient showed postoperative recovery of the nail plate and remains under outpatient follow-up due to the risk of recurrence (Fig. 4).

**Fig. 4:**
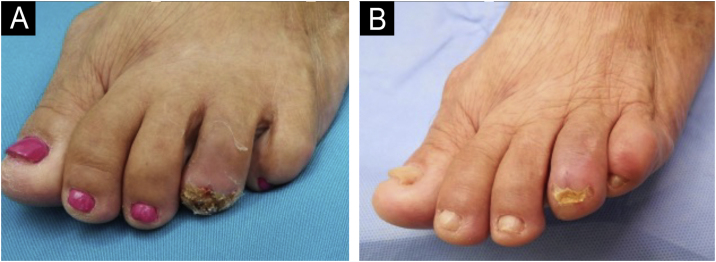
(A) 30-days postoperative: presence of crusts and scaling. (B) 90-days postoperative: complete healing with apparent recovery of the nail plate.

In approximately 36% of cases, SAF presents with erosive or lytic bone lesions.^4^, ^5^ Ultrasound examination can provide additional important information for surgical planning, such as tumor size, location, content, and the presence of vascularization on Doppler. It can also be used for monitoring recurrences.^6^

The treatment of choice is surgical excision, with a recurrence rate ranging from 10% to 24%, likely associated with incomplete resections. There are no reported cases of malignancy in the literature, and there is no consensus on surgical margins.^1^

The histopathological study reveals a non-encapsulated, moderately circumscribed lesion located in the dermis, which may extend into the hypodermis, fascia, or periosteal layer. There is a monomorphous proliferation of spindle cells resembling fibroblasts, embedded in a myxoid collagen stroma.^7^ A characteristic finding is the presence of fibrosis areas alternating with myxoid stroma. Nuclear atypia and mitotic figures are rare.^8^

In the immunohistochemical examination, tumor cells in SAF show immunoreactivity for CD34, EMA, and CD99. In the study by Fetsch et al.,^9^ which first described this tumor in 2001, positivity was reported as 91.3% for CD34, 72% for EMA, and 84.6% for CD99. Additionally, negativity is expected for cytokeratin, melanocytic markers, Smooth Muscle Actin (SMA), and desmin.^10^

The histopathological differential diagnoses include tumors with myxoid and fibromyxoid proliferation, including ungual fibroma, acquired digital fibrokeratoma, low-grade fibromyxoid sarcoma, myxoid dermatofibrosarcoma protuberans (DFSP), angiomyxoma and myxoid neurofibroma, which will be differentiated by immunohistochemical study.

CD34-positive tumors include DFSP, neurofibroma, and angiomyxoma. Myxoid neurofibroma has a neural appearance and is positive for S100. On the other hand, DFSP may have extensive myxoid areas, mimicking SAF, with positivity for CD34 and EMA, making it a difficult differential diagnosis.^1^ Peripheral areas, even if small, with the classic histological characteristics of DFSP, including a more infiltrative growth pattern, suggest this diagnosis.^9^

Superficial acral fibromyxoma (SAF) is a rare mesenchymal tumor with a challenging diagnosis, as it exhibits polymorphic clinical and dermoscopic features that resemble other digital lesions. An anatomopathological study and immunohistochemical examination are necessary for an accurate diagnosis. Despite its benign behavior, SAF can involve bone erosion and has high recurrence rates. In this context, Mohs surgery may be a good alternative for better margin control.

## Financial support

None declared.

## Authors’ contributions

Ana Carolina Baião Silva: Participated in generating data, literature review, and writing the paper.

Helena Maciel Guerra: Participated in generating data and approved the final version of this paper.

Leonardo Ávila: Participated in generating data and approved the final version of this paper.

Rafael Fantelli Stelini: Participated in generating data, writing the paper, and approved the final version of this paper.

Renata Ferreira Magalhães: Participated in writing the paper and approved the final version of this paper.

Laura Bertanha: Participated in generating data, writing the paper, reviewing the pertinent data, and approving the final version of this paper.

## Conflicts of interest

None declared.
